# Pharmacological HIF-PH Inhibition Suppresses Myoblast Differentiation Through Continued HIF-1α Stabilization †

**DOI:** 10.3390/ijms26115410

**Published:** 2025-06-05

**Authors:** Yuya Miki, Akinobu Ochi, Hideki Uedono, Yoshinori Kakutani, Mitsuru Ichii, Yuki Nagata, Katsuhito Mori, Yasuo Imanishi, Tetsuo Shoji, Tomoaki Morioka, Masanori Emoto

**Affiliations:** 1 Department of Metabolism, Endocrinology and Molecular Medicine, Osaka Metropolitan University Graduate School of Medicine, Osaka 545-8585, Japan; 2 Division of Internal Medicine, Inoue Hospital, Osaka 564-0053, Japan; 3 Department of Vascular Medicine, Osaka Metropolitan University Graduate School of Medicine, Osaka 545-8585, Japan; 4 Vascular Science Center for Translational Research, Osaka Metropolitan University Graduate School of Medicine, Osaka 545-8585, Japan; 5 Department of Nephrology, Osaka Metropolitan University Graduate School of Medicine, Osaka 545-8585, Japan

**Keywords:** HIF-PH inhibitor, HIF-1α stabilization, muscle differentiation

## Abstract

Hypoxia-inducible factor prolyl hydroxylase (HIF-PH) inhibitors continually stabilize hypoxia-inducible factor-1α (HIF-1α). These inhibitors are effective in the clinical treatment of renal anemia. However, the effects of continued HIF-1α stabilization on skeletal muscle differentiation remain unclear. This study aimed to investigate the effects of continued HIF-1α stabilization on skeletal muscle differentiation using a HIF-PH inhibitor in both in vitro and in vivo models. We cultured mouse C2C12 myoblasts to differentiate into myotubes with or without FG-4592, a HIF-PH inhibitor. Additionally, we treated nine-week-old male C57BL/6 mice with either FG-4592 or vehicle via intraperitoneal injections three times a week for four weeks. In vitro, FG-4592 treatment stabilized HIF-1α continually. Morphological analysis revealed that 72 h FG-4592 treatment suppressed differentiation of C2C12 myoblasts into myotubes. This treatment decreased the gene and protein expression of MyoD and myogenin, reduced the protein expression of myosin heavy chain (MHC), and increased the gene and protein expression of myostatin. HIF-1α knockdown mitigated the decrease in MHC protein expression induced by FG-4592. In vivo, FG-4592 treatment increased HIF-1α protein expression and decreased MyoD, myogenin, and MHC protein expression in gastrocnemius muscle. These findings suggest that pharmacological HIF-PH inhibition suppresses myoblast differentiation through continued HIF-1α stabilization.

## 1. Introduction

Sarcopenia, a condition characterized by the loss of skeletal muscle mass and decline in skeletal muscle function, becomes more prevalent as the chronic kidney disease (CKD) stage progresses [1]. Loss of skeletal muscle mass leads to reduced physical activity [2] and increased mortality [3]. As the CKD stage progresses, patients with sarcopenia have higher mortality than those without sarcopenia. Therefore, it is important to consider the effect of treatments for CKD patients on skeletal muscle mass. The prevalence of renal anemia also increases with the progression of CKD stages [4], commonly treated with erythropoiesis-stimulating agents (ESAs). However, ESA-resistant renal anemia patients, who do not achieve increased hemoglobin levels despite higher ESA doses, have higher mortality [5]. Recently, hypoxia-inducible factor prolyl hydroxylase (HIF-PH) inhibitors have shown potential to improve ESA-resistant renal anemia in clinical practice as an alternative treatment to ESAs. HIF-PH inhibitors suppress the activity of HIF-PH, thereby continually stabilizing the hypoxia-inducible factor-1α (HIF-1α) protein under normoxia, mimicking hypoxia [6]. Despite their potential benefits, the effect of continued HIF-1α stabilization by HIF-PH inhibitors on skeletal muscle mass remains unclear.

Hypoxia, which stabilizes the HIF-1α protein, both enhances muscle cell differentiation in the short term and reduces skeletal muscle mass in the long term. In humans, short-term hypoxia during resistance training enhances muscle cell differentiation more effectively than training under normoxia [7,8]. However, continued exposure to high altitudes leads to skeletal muscle mass reduction as an adaptive response to hypoxia [9]. Skeletal muscle mass reduction is also observed in patients with heart failure or chronic obstructive pulmonary disease, who often experience chronic hypoxia, compared to healthy individuals [10,11]. Thus, long-term hypoxia contributes to skeletal muscle mass reduction. Therefore, we hypothesized that continued HIF-1α stabilization by HIF-PH inhibitors could negatively affect skeletal muscle differentiation. We aimed to investigate the effects of continued HIF-1α stabilization on muscle differentiation using FG-4592 (roxadustat), a HIF-PH inhibitor.

## 2. Results

### 2.1. FG-4592 Stabilizes HIF-1α Protein Expression and Enhances Hypoxia-Response Element Activity in C2C12 Myoblasts

**Figure 1:**
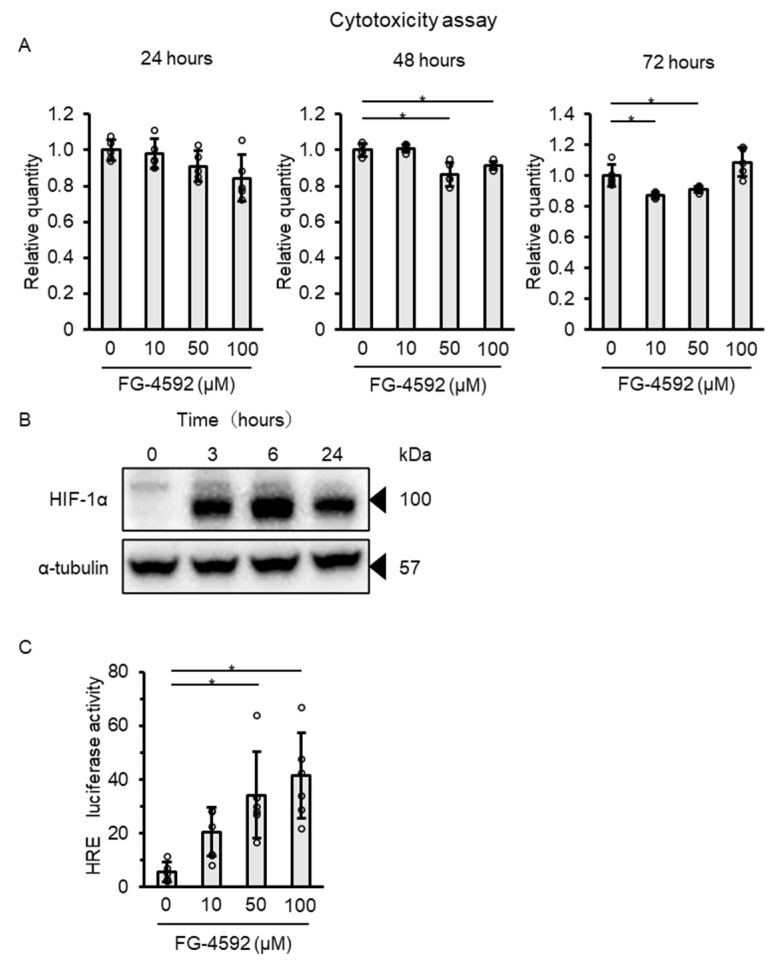
Effects of FG-4592 on C2C12 myoblasts: cytotoxicity, cell viability, HIF-1α expression, and HRE activity. (**A**) Cytotoxicity in C2C12 myoblasts treated with 0, 10, 50, or 100 µM FG-4592 for 24, 48, and 72 h. Means ± SD were calculated from five independent experiments. Circles show individual outcomes. Statistical significance was assessed using one-way ANOVA with post hoc Dunnett’s test (* *p* < 0.05). (**B**) HIF-1α expression in total cell lysates treated with 100 µM FG-4592 for 0, 3, 6, and 24 h, probed with HIF-1α and α-tubulin antibodies. (**C**) HRE activity in C2C12 myoblasts treated with 0, 10, 50, or 100 µM FG-4592 for 24 h, using a luciferase reporter assay. Means ± SD from five experiments. Circles represent individual outcomes. One-way ANOVA with Dunnett’s test (* *p* < 0.05). HRE: hypoxia-response element.

To investigate whether FG-4592 effectively stabilizes HIF-1α and activates hypoxia-response element (HRE), we first evaluated the effect of FG-4592 on HIF-1α protein expression in C2C12 myoblasts. After reaching confluence, the myoblasts were exposed to differentiation medium with concurrent FG-4592 treatment. FG-4592 at concentrations of 0, 10, 50, and 100 µM did not induce cytotoxicity after 24, 48, or 72 h (Figure 1A). FG-4592 treatment increased HIF-1α protein expression in the total cell lysate within three hours, with an elevated level persisting for 24 h (Figure 1B). In addition, HRE activity, assessed via a luciferase assay at 24 h after treatment, showed a dose-dependent increase when exposed to FG-4592 (Figure 1C).

### 2.2. FG-4592 Suppresses the Differentiation of C2C12 Myoblasts into Myotubes

**Figure 2:**
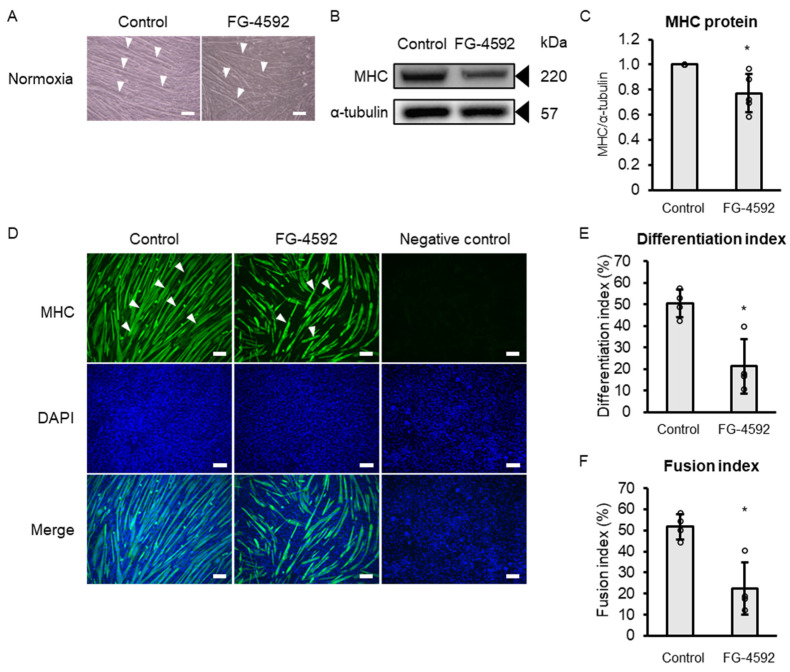
Effects of FG-4592 on C2C12 myoblast differentiation. (**A**) Light microscopy of C2C12 myoblasts after 72 h with vehicle (control) or 100 µM FG-4592 under normoxia. Arrowheads indicate representative myotubes. Scale bar: 100 µm. (**B**) Western blot of MHC in C2C12 myoblasts after 72 h with vehicle (control) or 100 µM FG-4592. Blots probed with MHC and α-tubulin antibodies. Representative results from four experiments. (**C**) Quantitative MHC expression normalized to α-tubulin and expressed as ratio to control. Means ± SD from four experiments. Circles represent individual outcomes. Unpaired Student’s *t*-test (* *p* < 0.05). (**D**) Fluorescent immunostaining images showing MHC positive C2C12 myotubes (green, arrowheads) and nuclei (blue) after 72 h with vehicle (control, (**left)**) or 100 µM FG-4592 (**middle**). Staining for MHC (**upper**), DAPI (**middle**), and merged images (**lower**). Negative control on the right. Scale bar: 100 µm. (**E**) Differentiation index after 72 h with 100 µM FG-4592. Index calculated as nuclei in myotubes to total nuclei in 10 fields per well. Means ± SD from four experiments. Circles represent individual outcomes. Unpaired Student’s *t*-test (* *p* < 0.05). (**F**) Fusion index after 72 h with 100 µM FG-4592. Index calculated as nuclei in myotubes with more than two nuclei in 10 fields per well to total nuclei. Means ± SD from four experiments. Circles represent individual outcomes. Unpaired Student’s *t*-test (* *p* < 0.05). DAPI—4′,6-diamidino-2-phenylindole; MHC—myosin heavy chain.

To determine whether FG-4592 suppresses muscle differentiation, we assessed the effect of FG-4592 on C2C12 myoblast differentiation into myotubes after a 72 h treatment period using morphological methods. Representative light microscopy images illustrate myotubes with elongated tubular structures (indicated by arrowheads) in the control group, whereas such structures appeared to be reduced in number and less developed in the FG-4592-treated group (Figure 2A). FG-4592 treatment decreased myosin heavy chain (MHC) protein expression (Figure 2B,C). Fluorescence immunostaining for MHC showed fewer visible myotubes in the FG-4592-treated cells compared to controls (Figure 2D). Quantitative analysis revealed significantly reduced differentiation and fusion index values for FG-4592-treated cells compared with controls (Figure 2E,F).

### 2.3. Concentration-Dependent Effects of FG-4592 on Muscle Differentiation-Related Markers

**Figure 3:**
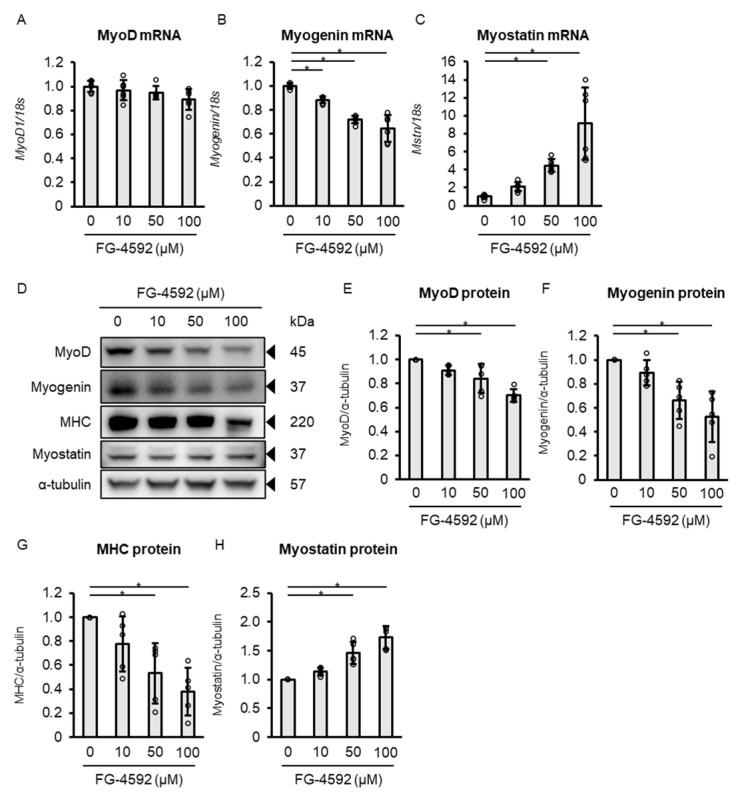
Effects of FG-4592 dosage on gene and protein expression in C2C12 myotubes. (**A**–**D**) Quantitative PCR analysis of gene expression in C2C12 myotubes treated with FG-4592 (0, 10, 50, 100 µM) for 72 h. Gene levels of MyoD (**A**), myogenin (**B**), and myostatin (**C**) were normalized to 18S rRNA. Means ± SD from six experiments. Circles represent individual data. One-way ANOVA with Dunnett’s test (* *p* < 0.05). (**D**) Western blot of MyoD, myogenin, MHC, and myostatin in C2C12 myotubes treated with FG-4592 (0, 10, 50, 100 µM) for 72 h. Blots probed with specific antibodies and α-tubulin as a loading control. Representative blots from five experiments. (**E**–**H**) Quantitative protein assessment in C2C12 myotubes treated with FG-4592 (0, 10, 50, 100 µM) for 72 h. Protein levels of MyoD (**E**), myogenin (**F**), MHC (**G**), and myostatin (**H**) normalized to α-tubulin and expressed as ratios to control (0 µM). Means ± SD from five experiments. One-way ANOVA with Dunnett’s test (* *p* < 0.05). MHC: myosin heavy chain.

To further clarify the molecular changes underlying the observed suppression of muscle differentiation, we analyzed dose-dependent changes in gene and protein expression associated with muscle differentiation in C2C12 myoblasts treated with 0, 10, 50, or 100 µM FG-4592 for 72 h. Gene expression analysis at 72 h following FG-4592 treatment showed no significant change in MyoD (Figure 3A), a dose-dependent decrease in myogenin (Figure 3B), and an increase in myostatin (Figure 3C). Protein expression analysis revealed dose-dependent decreases in MyoD, myogenin, and MHC, and an increase in myostatin (Figure 3D–H).

### 2.4. Time-Course Analysis of Effects of FG-4592 on Markers Related to Muscle Differentiation

**Figure 4:**
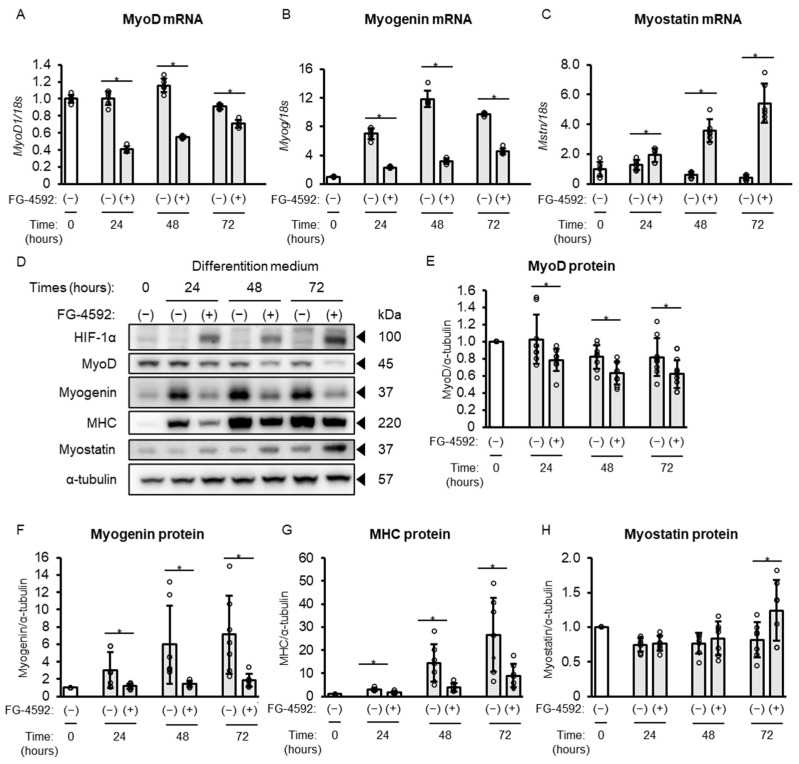
Time-dependent effects of FG-4592 on gene and protein expression in C2C12 myoblasts. (**A**–**D**) Quantitative PCR analysis of MyoD (**A**), myogenin (**B**), and myostatin (**C**) in C2C12 myoblasts at 0, 24, 48, and 72 h. Myoblasts were cultured in differentiation medium with 100 µM FG-4592 or vehicle. Gene expression was normalized to 18S rRNA. Means ± SD from six experiments. Circles represent individual data. Unpaired Student’s *t*-test (* *p* < 0.05). (**D**) Western blot of MyoD, myogenin, and myostatin in C2C12 myoblasts at 0, 24, 48, and 72 h. Blots probed with specific antibodies and α-tubulin as a loading control. Representative results from more than seven experiments. (**E**–**H**) Quantitative protein assessments in C2C12 myoblasts at 0, 24, 48, and 72 h. Protein levels of MyoD (**E**), myogenin (**F**), MHC (**G**), and myostatin (**H**) normalized to α-tubulin and expressed as fold change relative to the 0 h time point. Means ± SD from more than seven experiments. Circles represent individual data. Unpaired Student’s *t*-test (* *p* < 0.05). MHC: myosin heavy chain.

To understand the temporal dynamics of FG-4592’s effects on differentiation, we performed a time-course analysis of both mRNA and protein levels for myogenic markers over 72 h of treatment with 100 µM FG-4592 in C2C12 myoblasts. Gene expression analysis revealed decreased expression levels of MyoD and myogenin, and increased expression of myostatin from 24 to 72 h following FG-4592 treatment (Figure 4A–C). More detailed analysis of the protein expression of MyoD, myogenin, and MHC showed decreases at 24, 48, and 72 h (Figure 4D–G), while that of myostatin was unchanged at 24 and 48 h, and then increased at 72 h after FG-4592 treatment (Figure 4D,H).

### 2.5. FG-4592 Suppresses Differentiation of C2C12 Myoblasts into Myotubes via HIF-1α Stabilization

**Figure 5:**
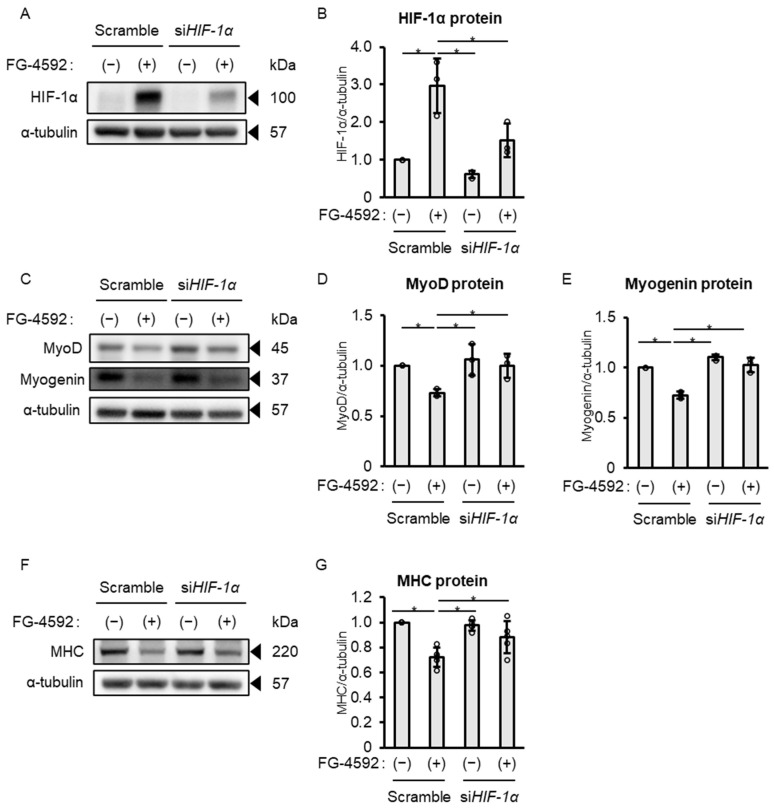
HIF-1α knockdown attenuates the FG-4592’s suppressive effect on MyoD, myogenin, and MHC protein expression in C2C12 myoblasts. (**A**) Western blot of HIF-1α in C2C12 myoblasts transfected with si*HIF-1α* or scrambled siRNA, treated with 100 µM FG-4592 or vehicle for 24 h. Blots probed with HIF-1α and α-tubulin antibodies. Representative blots from three experiments. (**B**) Quantitative HIF-1α protein expression in C2C12 myoblasts transfected with si*HIF-1α* or scrambled siRNA, treated with 100 µM FG-4592 or vehicle for 24 h. HIF-1α normalized to α-tubulin and expressed as ratio to control (scrambled siRNA + vehicle). Means ± SD from three experiments. One-way ANOVA with Tukey’s test (* *p* < 0.05). (**C**) Western blot of MyoD and myogenin in C2C12 myoblasts transfected with si*HIF-1α* or scrambled siRNA, treated with 100 µM FG-4592 or vehicle for 24 h. Blots probed with MyoD, myogenin, and α-tubulin antibodies. Representative blots from three experiments. (**D**,**E**) Quantitative MyoD (**D**) and myogenin (**E**) protein expression in C2C12 myoblasts transfected with si*HIF-1α* or scrambled siRNA, treated with 100 µM FG-4592 or vehicle for 24 h. MyoD and myogenin normalized to α-tubulin and expressed as ratio to control (scrambled siRNA + vehicle). Means ± SD from five experiments. One-way ANOVA with Tukey’s test (* *p* < 0.05). (**F**) Western blot of MHC in C2C12 myoblasts transfected with si*HIF-1α* or scrambled siRNA, treated with 100 µM FG-4592 or vehicle for 24 h. Blots probed with MHC and α-tubulin antibodies. Representative blots from five experiments. (**G**) Quantitative MHC protein expression in C2C12 myoblasts transfected with si*HIF-1α* or scrambled siRNA, treated with 100 µM FG-4592 or vehicle for 24 h. MHC normalized to α-tubulin and expressed as ratio to control (scrambled siRNA + vehicle). Means ± SD from five experiments. One-way ANOVA with Tukey’s test (* *p* < 0.05). MHC: myosin heavy chain.

To elucidate the mechanism related to the suppressive effect of FG-4592 on differentiation in C2C12 myoblasts, the role of HIF-1α was investigated using siRNA. Under vehicle-treated conditions (without FG-4592), C2C12 myoblasts transfected with si*HIF-1α* showed reduced HIF-1α protein expression at 24 h. Transfection with si*HIF-1α* significantly attenuated the FG-4592-induced increase in HIF-1α protein expression at 24 h (Figure 5A,B). Knockdown of HIF-1α attenuated FG-4592-induced suppression of MyoD and myogenin protein expression (Figure 5C–E). The suppressive effect of FG-4592 on MHC protein expression was partially reversed by si*HIF-1α* after 24 h (Figure 5F,G). These results indicate that the suppressive effect of FG-4592 on C2C12 myoblast differentiation was mediated by continued HIF-1α stabilization.

### 2.6. Effects of FG-4592 on Differentiation of C2C12 Myoblasts Similar to Those of Hypoxic Treatment

**Figure 6:**
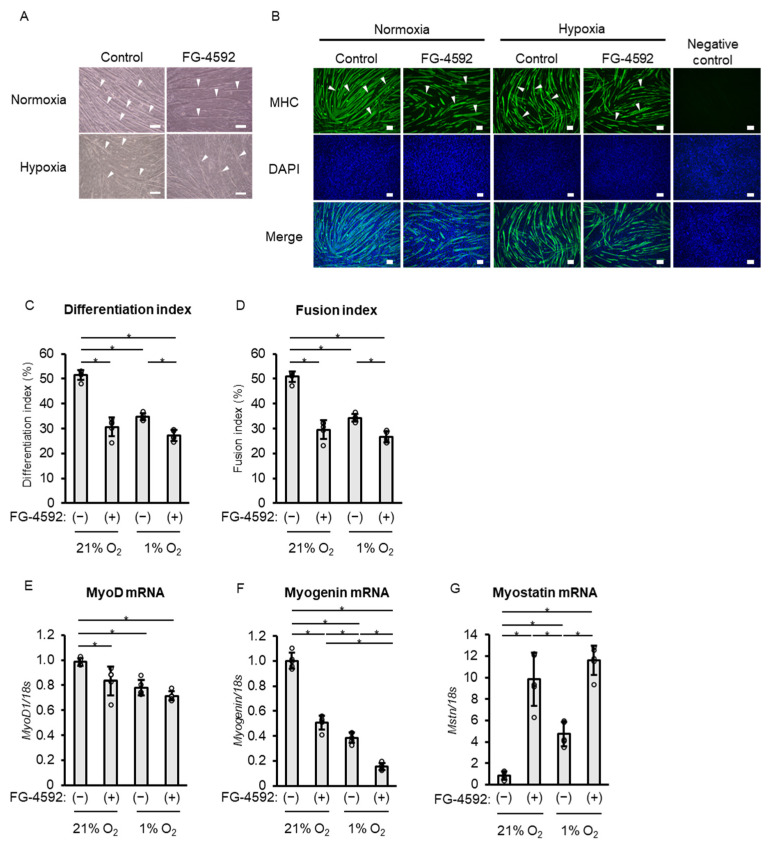
Comparison of suppressive effects on C2C12 myoblast differentiation: FG-4592 vs. hypoxia. (**A**) Light microscopy of C2C12 myoblasts after 72 h with 100 µM FG-4592 or vehicle under normoxia (21% O_2_, (**top**)) and hypoxia (1% O_2_, (**bottom**)). Arrowheads indicate representative myotubes. Scale bar: 100 µm. (**B**) Fluorescent immunostaining images showing MHC positive C2C12 myotubes (green, arrowheads) and nuclei (blue) after 72 h with vehicle (control) or 100 µM FG-4592 under normoxia (21% O_2_) and hypoxia (1% O_2_). Staining for MHC (**upper**), DAPI (**middle**), and merged images (**lower**). Negative control on the right. Scale bar: 100 µm. (**C**) Differentiation index after 72 h with or without 100 µM FG-4592 under normoxia (21% O_2_) and hypoxia (1% O_2_). Index calculated as nuclei in myotubes to total nuclei in 10 fields per well. Means ± SD from five experiments. Circles represent individual outcomes. One-way ANOVA with Tukey’s test (* *p* < 0.05). (**D**) Fusion index after 72 h with or without 100 µM FG-4592 under normoxia (21% O_2_) and hypoxia (1% O_2_). Index calculated as nuclei in myotubes with more than two nuclei in 10 fields per well to total nuclei. Means ± SD from five experiments. Circles represent individual outcomes. One-way ANOVA with Tukey’s test (* *p* < 0.05). (**E**–**G**) Quantitative PCR analysis of MyoD (**E**), myogenin (**F**), and myostatin (**G**) in C2C12 myotubes treated with 100 µM FG-4592 or vehicle under 21% or 1% O_2_ for 72 h. Gene expression of MyoD, myogenin, and myostatin normalized to 18S rRNA. Means ± SD from five experiments. One-way ANOVA with Tukey’s test (* *p* < 0.05). DAPI—4′,6-diamidino-2-phenylindole; MHC—myosin heavy chain.

To determine whether the suppressive effect of FG-4592 on differentiation in C2C12 myoblasts is similar to that of hypoxia, we cultured C2C12 myoblasts under the following conditions: a normoxia (21% O_2_) vehicle group (normoxia–vehicle group), a normoxia group with 100 µM FG-4592 (normoxia–FG-4592 group), a hypoxia (1% O_2_) vehicle group (hypoxia–vehicle group), and a hypoxia group with 100 µM FG-4592 (hypoxia–FG-4592 group). Light microscopy and fluorescence immunostaining images are presented in Figure 6A,B. Differentiation index and fusion index were significantly decreased in the hypoxia–vehicle group compared to the normoxia–vehicle group. No significant differences were observed between the normoxia–FG-4592 group and the hypoxia–vehicle group. However, in the hypoxia–FG-4592 group, both indices were further significantly decreased compared to the hypoxia–vehicle group (Figure 6C,D). When compared to the normoxia–vehicle group, all other groups—the normoxia–FG-4592 group, the hypoxia-vehicle group, and the hypoxia–FG-4592 group—showed a decrease in MyoD and myogenin gene expression, and an increase in myostatin gene expression (Figure 6E–G). However, the extent of myogenin gene expression downregulation was significantly greater in the hypoxia–vehicle group than in the normoxia–FG-4592 group (Figure 6F). The upregulation of myostatin gene expression was significantly more pronounced in the normoxia–FG-4592 group than in the hypoxia–vehicle group (Figure 6G).

Next, we examined changes in gene expression in C2C12 myoblasts treated with FG-4592 under hypoxia. MyoD gene expression showed no significant change with or without FG-4592 treatment. However, myogenin gene expression in the hypoxia–FG-4592 group was significantly lower, and myostatin gene expression was significantly higher compared to the hypoxia–vehicle group (Figure 6E–G).

### 2.7. FG-4592 Suppresses Skeletal Muscle Differentiation In Vivo

**Figure 7:**
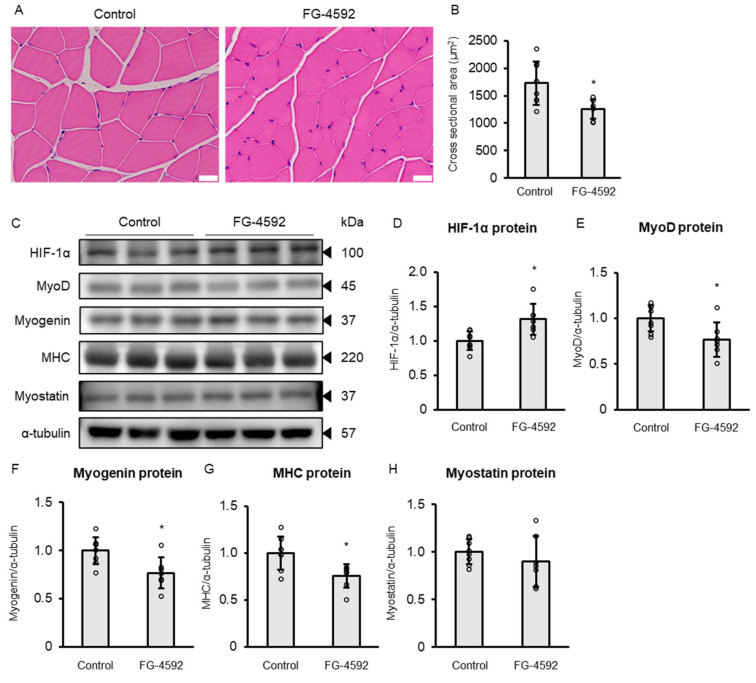
Effects of FG-4592 on skeletal muscle in vivo. (**A**) Light microscopy of gastrocnemius muscle of C57BL/6 mice treated with FG-4592 or vehicle three times weekly for four weeks. Scale bar: 20 µm. (**B**) Comparison of the cross-sectional area of the muscle fibers in the gastrocnemius muscle of C57BL/6 mice treated with 100 µM FG-4592 or vehicle three times weekly for four weeks. Means ± SD from FG-4592 (*n* = 7) and control (*n* = 8) groups. Circles show individual data. Unpaired Student’s *t*-test (* *p* < 0.05). (**C**) Western blot of HIF-1α, MyoD, myogenin, MHC, and myostatin in gastrocnemius muscles of C57BL/6 mice treated with FG-4592 or vehicle three times weekly for four weeks. Blots probed with specific antibodies and α-tubulin as a loading control. Representative blots from three experiments. (**D**–**H**) Quantitative protein assessment of HIF-1α (**D**), MyoD (**E**), myogenin (**F**), MHC (**G**), and myostatin (**H**) in gastrocnemius muscles of mice treated with FG-4592 (*n* = 7) or vehicle (Control, *n* = 8), three times weekly for four weeks. Protein levels normalized to α-tubulin and expressed as ratio to control. Means ± SD from FG-4592 (*n* = 7) and control (*n* = 8) groups. Circles show individual data. Unpaired Student’s *t*-test (* *p* < 0.05). MHC: myosin heavy chain.

To assess whether the suppressive effects of FG-4592 on myotube formation observed in vitro also occur in vivo, we administered FG-4592 to mice and analyzed skeletal muscle tissue for changes in fiber morphology and myogenic marker expression. C57BL/6 mice were administered intraperitoneal injections of FG-4592 or vehicle control, three times per week for four weeks. Table 1 presents the body weight and lower limb muscle weights. FG-4592 treatment did not significantly reduce body weight or the weights of the gastrocnemius, extensor digitorum longus, or soleus muscles. However, histological analysis revealed smaller cross-sectional areas in gastrocnemius muscle fibers in FG-4592-treated mice (Figure 7A,B). Subsequently, Western blot analysis was performed on gastrocnemius muscle samples. HIF-1α protein expression in the gastrocnemius muscle was increased in the FG-4592 group (Figure 7C,D). In contrast, protein levels of MyoD, myogenin, and MHC were significantly reduced in the FG-4592 group (Figure 7C,E–G). However, myostatin protein expression showed no significant difference between the groups (Figure 7C,H). These results suggest that four weeks of FG-4592 treatment suppresses skeletal muscle differentiation in vivo.

## 3. Discussion

The present findings demonstrated that continued HIF-1α protein stabilization by FG-4592 results in suppression of differentiation of C2C12 myoblasts into myotubes. Notably, FG-4592 reduced the expression of the key myogenic factors MyoD and myogenin, while it concurrently elevated the level of myostatin. Moreover, si*HIF-1α* transfection mitigated FG-4592-induced suppression of MHC protein expression in C2C12 myoblasts, indicating a direct role of HIF-1α stabilization in this process. Consistent with these observations, intraperitoneal administration of FG-4592 in mice, conducted three times weekly over a period of four weeks, led to the decreased cross-sectional area of muscle fibers and the decreased expression levels of MyoD, myogenin, and MHC proteins in gastrocnemius muscle.

Our study demonstrated that treatment with FG-4592 significantly suppressed the gene and protein expression of MyoD and myogenin both in vitro and in vivo. Knockdown of HIF-1α attenuated negative effects by FG-4592 on MyoD, myogenin, and MHC protein expression. Previous studies have shown that prolonged hypoxia decreases the protein expression of MyoD and myogenin and suppresses muscle differentiation in vitro [12,13]. The downregulation of HIF-1α attenuates the negative effects of hypoxia on MyoD and myogenin protein expression [12]. Cobalt chloride, which mimics hypoxia by stabilizing HIF-1α, also suppresses MyoD and myogenin expression and leads to the suppression of muscle differentiation [14]. Our results are consistent with these findings and suggest that continued HIF-1α stabilization by FG-4592 suppresses MyoD and myogenin protein expression, similar to the effects of prolonged hypoxia.

This study also revealed that FG-4592 treatment elevates both the gene and protein expression of myostatin. The myostatin gene promoter in rat cardiomyocytes contains an HRE sequence [15], and thus it is plausible that continued HIF-1α stabilization by FG-4592 could directly augment myostatin expression by interacting with this HRE sequence. Myostatin downregulates MyoD and myogenin protein expression through Smad3 in C2C12 cells [16,17]. However, in this study, the increase in myostatin protein expression occurred later than decreases in MyoD and myogenin protein expression. Thus, our findings indicate that the increase in myostatin protein may occur independently of the decreases in MyoD and myogenin protein levels.

This is the first report to document suppression of myoblast differentiation by continued HIF-1α stabilization using an HIF-PH inhibitor. These findings complement those reported by Ichii et al., who found a decrease in serum creatinine level in patients undergoing hemodialysis with roxadustat (FG-4592) treatment, which was reversed upon discontinuation of roxadustat [18]. Serum creatinine levels are known to have a correlation with skeletal muscle mass in hemodialysis patients [19]. Therefore, our findings may explain these observed alterations in serum creatinine concentrations.

This study has several limitations. First, though FG-4592 suppresses differentiation of C2C12 myoblasts into myotubes, the underlying mechanisms were not fully elucidated. Second, while si*HIF-1α* transfection partially reversed suppression of MHC protein expression caused by FG-4592, it did not lead to complete recovery. This suggests that pathways other than HIF-1α may be involved in the suppressive effect of FG-4592 on C2C12 myoblast differentiation. Third, under hypoxic conditions (1% O_2_) combined with FG-4592 treatment, we observed a decrease in MyoD gene expression and an increase in myostatin gene expression compared to hypoxia alone, indicating that FG-4592 may suppress differentiation through mechanisms independent of HIF-1α. Fourth, while our in vivo experiments were conducted over a four-week period, FG-4592 significantly reduced muscle differentiation markers. However, the muscle mass remained unchanged after the four-week treatment. Thus, the long-term effects of FG-4592 on skeletal muscle mass remain unclear and warrant further investigation. Fifth, we did not employ cold injury or cardiotoxin-induced injury models, which are typically utilized to evaluate skeletal muscle differentiation in vivo, since our primary aim was to examine the effects of long-term clinical use of FG-4592 administration on differentiation markers. Therefore, future studies investigating the effects of long-term FG-4592 treatment should incorporate these injury models to confirm and extend our findings. Finally, while we observed an increase in myostatin protein expression following FG-4592 treatment, its precise role in myoblast differentiation remains unclear. Future studies should explore the precise role of myostatin in the suppression of myoblast differentiation.

Our findings indicate that pharmacological HIF-PH inhibition suppresses myoblast differentiation through continued HIF-1α stabilization. Further understanding of the role of continued HIF-1α stabilization in skeletal muscle differentiation is crucial for ensuring the safety of patients prescribed HIF-PH inhibitors related to maintenance of skeletal muscle mass.

## 4. Materials and Methods

### 4.1. Cell Culture and Differentiation

We acquired mouse C2C12 myoblasts from the American Type Culture Collection (Manassas, VA, USA) and used them within 15 passages. They were cultured at 37 °C in an atmosphere with 5% CO_2_ in proliferation medium, consisting of Dulbecco’s Modified Eagle Medium (DMEM) (Nacalai Tesque, Kyoto, Japan) containing 10% fetal bovine serum (FBS) (Thermo Fisher Scientific, Waltham, MA, USA), as well as 100 U/mL penicillin with 100 μg/mL streptomycin (Nacalai Tesque, Kyoto, Japan). For differentiation, we incubated confluent myoblasts in differentiation medium consisting of DMEM supplemented with 3% horse serum (Thermo Fisher Scientific) and the same antibiotics. We cultured C2C12 myoblasts in differentiation medium with or without FG-4592 (MedChemExpress, Monmouth Junction, NJ, USA) for up to 72 h, replacing the medium every 24 h.

### 4.2. Cytotoxicity Assay

We assessed cytotoxicity of FG-4592 using the CellTox™ Green Cytotoxicity Assay (Promega, Madison, WI, USA) following the manufacturer’s protocol. C2C12 cells were seeded into 96-well plates, then treated with FG-4592 for 24, 48, or 72 h. We added CellTox™ Green Reagent and determined the fluorescence at an excitation wavelength of 485 nm and emission wavelength of 530 nm using a Varioskan™ LUX Multimode Microplate Reader (Thermo Fisher Scientific).

### 4.3. Transfection and Dual Luciferase Reporter Assay

HIF-1α translocates into the nucleus and exerts its biological activity by binding to the HRE. Therefore, we measured the HRE activity induced by FG-4592. We transfected C2C12 myoblasts at 70% confluence using Lipofectamine™ LTX Reagent with PLUS™ Reagent (Thermo Fisher Scientific). We introduced 600 ng of pGL4.42[*luc2P*/HRE/Hygro] firefly luciferase reporter plasmid (E400A, Promega) and 20 ng of pRL-null Vector (E2271, Promega). After 24 h of FG-4592 treatment, we measured luciferase activity with a Dual-Luciferase Reporter Assay System (Promega) and Wallac ARVO SX 1420 luminometer (PerkinElmer, Waltham, MA, USA).

### 4.4. Western Blot Analysis

We performed Western blot analysis as previously described [20]. Primary antibodies included anti-HIF-1α (GTX127309, GeneTex, Irvine, CA, USA) at 1:3000, anti-MyoD (sc-377460, Santa Cruz Biotechnology, Dallas, TX, USA) at 1:500, anti-myogenin (sc-12732, Santa Cruz Biotechnology) at 1:500, anti-myosin heavy chain (MAB4470, R&D Systems, Minneapolis, MN, USA) at 1:1000, anti-myostatin (19142-1-AP, Proteintech, Rosemont, IL, USA) at 1:2000, and anti-α-tubulin (ab40742, Abcam, Cambridge, UK) at 1:10,000. Secondary antibodies were anti-mouse (sc-2005, Santa Cruz Biotechnology) at 1:5000 and anti-rabbit (6440-05, SouthernBiotech, Birmingham, AL, USA) at 1:5000. Detection was performed using a FUSION Solo S system (Vilber, Collégien, France).

### 4.5. Real-Time Quantitative PCR

We performed real-time quantitative PCR with an Applied Biosystems 7500 Fast Real-Time PCR System (Applied Biosystems, Waltham, MA, USA) using the comparative cycle threshold method with 18S ribosomal RNA for normalization. TaqMan primer-probe sets for MyoD (*Myod1,* Mm00440387_m1), myogenin (*Myog*, Mm00446194_m1), myostatin (*Mstn*, Mm01254559_m1), as well as 18S rRNA Control Mix, were used (Applied Biosystems).

### 4.6. Immunofluorescence Staining

We fixed C2C12 myotubes with 4% paraformaldehyde. We incubated them overnight at 4 °C with anti-MHC antibody (MAB4470, R&D Systems) at 1:200. Then, we treated them with Alexa Fluor™ anti-mouse IgG (A21202, Invitrogen, Waltham, MA, USA) at 1:200. Nuclei were counterstained with DAPI (Vector Laboratories, Burlingame, CA, USA). Fluorescence microscopy (BZ-X810, KEYENCE, Osaka, Japan) was used for the examinations. The differentiation and fusion indices were calculated using ImageJ (ver. 1.54g, NIH) across 10 random fields. Differentiation index was the number of nuclei in myotubes divided by total nuclei; fusion index was the number of nuclei in myotubes with more than two nuclei divided by total nuclei [21].

### 4.7. siHIF-1α Transfection

When C2C12 myoblasts reached approximately 80% confluency, we transfected them with small interfering RNA targeting HIF-1α (si*HIF-1α*) (Santa Cruz Biotechnology, Dallas, TX, USA) or scrambled siRNA (Santa Cruz Biotechnology) using Lipofectamine™ RNAiMAX (Invitrogen). After 24 h, we treated cells with or without FG-4592.

### 4.8. Animal Experiments

Male C57BL/6 mice, aged nine weeks, were acquired from Japan SLC (Shizuoka, Japan). All experimental procedures adhered to the National Institutes of Health Guide for the Care and Use of Laboratory Animals and were approved by the Animal Ethics Committee of Osaka Metropolitan University (approval number 23053). We prepared FG-4592 in a solution of 5% Dimethyl sulfoxide, 5% Tween 80, 40% polyethylene glycol 300, and 50% distilled water. Each mouse received an intraperitoneal injection of FG-4592 at 10 mg/kg of body weight or the vehicle three times weekly for four weeks. We harvested the gastrocnemius muscle tissues of mice on day 31.

Based on previous reports that evaluated muscle cross-sectional area using hematoxylin and eosin (HE)-stained sections [22,23], we measured cross-sectional area from HE-stained gastrocnemius muscle tissue. Paraffin sections were prepared from the central region of harvested gastrocnemius muscles. The sections were stained with HE and observed with the bright-field mode of a fluorescence microscope (BZ-X810, KEYENCE, Osaka, Japan). The cross-sectional areas of muscle fibers were measured and analyzed using ImageJ software (ver. 1.54g, NIH) across 10 randomly selected, non-overlapping fields per sample.

### 4.9. Statistical Analysis

We statistically analyzed data from at least three independent experiments using Student’s *t*-test and one-way ANOVA with post hoc Tukey’s test or Dunnett’s test. Results were expressed as mean ± standard deviation (SD). A *p*-value < 0.05 was considered statistically significant. Analyses were conducted using EZR (version 1.61, Saitama Medical Center, Jichi Medical University, Saitama, Japan), a graphical interface for R (version 4.2.2, The R Foundation for Statistical Computing, Vienna, Austria) [24].

## Figures and Tables

**Table 1:** Body weight and lower limb muscle weights after four weeks of FG-4592 treatment in mice.

	Control (*n* = 8)	FG-4592 (*n* = 7)	*p*-Value
Body weight (g)	26.38 ± 1.22	25.99 ± 1.50	0.586
Gastrocnemius muscle (mg)	318.28 ± 28.59	297.69 ± 42.25	0.313
Extensor digitorum longus muscle (mg)	23.34 ± 0.93	22.54 ± 1.90	0.283
Soleus muscle (mg)	19.33 ± 2.68	19.54 ± 1.91	0.861

The data are presented as mean ± standard deviation (SD) and the *p*-values. The *p*-values were obtained using Student’s *t*-test.

## Data Availability

The data that support the findings of this study are available from the corresponding author (A.O.) upon reasonable request.

## Abbreviations

The following abbreviations are used in this manuscript:

CKD	Chronic kidney disease
ESA	Erythropoiesis-stimulating agent
HIF-PH	Hypoxia-inducible factor prolyl hydroxylase
HIF-1α	Hypoxia-inducible factor-1α
DMEM	Dulbecco’s Modified Eagle Medium
FBS	Fetal bovine serum
HRE	Hypoxia-response element
MHC	Myosin heavy chain
SD	Standard deviation
